# HMGA1 promotes gastric cancer growth and metastasis by transactivating SUZ12 and CCDC43 expression

**DOI:** 10.18632/aging.203130

**Published:** 2021-06-24

**Authors:** Qiong Yang, Yusi Wang, Mengshu Li, Zhi Wang, Jieming Zhang, Weiyu Dai, Miaomiao Pei, Linjie Hong, Yizhi Xiao, Hongsong Hu, Jiaying Li, Jianjiao Lin, Xiaosheng Wu, Yaying Chen, Miaojuan Huang, Aimin Li, Side Liu, Weimei Tang, Li Xiang, Jide Wang

**Affiliations:** 1 Guangdong Provincial Key Laboratory of Gastroenterology, Department of Gastroenterology, Nanfang Hospital, Southern Medical University, Guangzhou 510515, China; 2 Department of Gastroenterology, Longgang District People’s Hospital, Shenzhen 518172, China; 3 Department of Gastroenterology, The Third Affiliated Hospital of Guangzhou Medical University, Guangzhou 510150, China; 4 The Second Affiliated Hospital of University of South China, Hengyang 421001, China

**Keywords:** HMGA1, SUZ12, CCDC43, gastric cancer, metastasis

## Abstract

HMGA1 protein is an architectural transcription factor that has been implicated in the progression of multiple malignant tumors. However, the role of HMGA1 in the growth and metastasis of gastric cancer (GC) has not yet been elucidated. Here, we show that HMGA1 is overexpressed in GC cells and the high expression of HMGA1 was correlated with worse survival in GC patients using a bioinformatics assay. Functionally, HMGA1 affected the EdU incorporation, colony formation, migration and invasion of GC cells by exogenously increasing or decreasing the expression of HMGA1. Mechanistically, HMGA1 directly bound to the SUZ12 and CCDC43 promoter and transactivated its expression in GC cells. Inhibition of SUZ12 and CCDC43 attenuated the proliferation, migration and invasiveness of HMGA1-overexpressing GC cells *in vitro*. Moreover, both HMGA1 and SUZ12/CCDC43 were highly expressed in cancer cells but not in normal gastric tissues, and their expressions were positively correlated. Finally, a tail vein metastatic assay showed that HMGA1 promoted SUZ12/CCDC43-mediated GC cell metastasis *in vivo*. Our findings suggest that HMGA1 promotes GC growth and metastasis by transactivating SUZ12 and CCDC43 expression, highlighting HMGA1 as a potential prognostic biomarker in the treatment of GC.

## INTRODUCTION

Gastric cancer (GC) is currently the fourth most common type of cancer worldwide and is the second cause of cancer-related deaths, with 738,000 deaths occurring every year across the globe [1, 2]. Over the past three decades, the incidence of gastric cancer has gradually declination as a result of improved treatment. Nevertheless, the molecular mechanisms underlying of GC invasion and metastasis have not been elucidated yet.

The HMGA1a and HMGA1b proteins were encoded by the high mobility group A1 (HMGA1) gene, which were produced through differential splicing of the same premessenger RNA. The other 11 internal amino acids located at the upstream of the second AT hook is the main difference between HMGA1a and HMGA1b [3, 4]. The biological meaning of these two diverse isoforms is not illuminated yet, due to functional studies indicated several overlapping roles. This gene is a transcription factor that binds to AT-rich sequences of DNA to regulate transcription, acting as a co-activator or co-repressor of gene expression [5, 6]. Studies have shown that HMGA1 promotes matrix metalloproteinase 2 (MMP2) transcription via directly binding to and advancing MMP2 promoter activity [7, 8]. Moreover, it was reported that expression of NUMB was negatively regulated by HMGA1 at the transcriptional and post-transcriptional levels in glioblastoma stem cells [9]. HMGA1 is strongly expressed during embryogenesis and in virtually all aggressive human cancers but is silenced in adult, differentiated tissues [9–13]. For example, the levels of HMGA1 was highly expressed in breast cancer tissues. [10]. Recent studies have shown that HMGA1 contributes to tumorigenesis in GC cancers [11]. Nevertheless, the specific function of HMGA1 in GC remains unclear.

The SUZ12 gene is located on chromosome 17 at q11.2 and encodes a protein consisting of 739 amino acid residues (https://www.ncbi.nlm.nih.gov/CCDS/CcdsBrowse.cgi). SUZ12 is difficult to detect in normal tissues but is amplified and overexpressed in several solid cancers, such as breast [14], GC [15] and head and neck squamous cell cancer (HNSCC) [16]. Moreover, SUZ12 knock- down induces impaired tumor growth, invasion and metastasis in bladder [17], gastric [15] and colorectal cancers [18]. Furthermore, we and others have found that SUZ12 promotes tumor cell epithelial-to-mesenchymal transition (EMT), which presents the critical function in the metastatic development of human carcinomas [19, 20].

The coiled-coil domain, a structural motif protein, was verified to be involved in various biological processes including mediation of gene expression, cell division, and membrane fusion [21–23]. Ectopic expression of the proteins in papillary thyroid carcinoma [23], lung cancer [24], cervical cancer [25], esophageal squamous cell carcinoma [26], pancreatic cancer [27] and ovarian cancer [28] has been shown to be related with the malignant behavior of human cancers. CCDC43 is a new member of this family located at chromosome 17q21.31 and consists of 224 amino acids (https://www.ncbi.nlm.nih.gov/gene/124808). We have implicated CCDC43 as an oncogenic factor in gastrointestinal cancers [29, 30]. Overexpression expression of CCDC43 protein promoted proliferation in GC and CRC. Furthermore, CCDC43 stimulated EMT, tumor invasion and metastasis. Therefore, CCDC43 serves a vital function in cancer onset and development.

In present work, we provided evidence that upregulation of HMGA1 increases the proliferative ability and migrative capacity of GC cell. Furthermore, HMGA1 promotes GC growth and metastasis by transactivating SUZ12 and CCDC43 expression. Therefore, the HMGA1-SUZ12/CCDC43 signaling axis may lead to GC onset and development.

## MATERIALS AND METHODS

### Cells lines

The immortalized normal gastric epithelial cell line GES-1 and seven human GC cell lines, including HGC-27, MKN-28, BGC-823, SGC-7901, MKN-45, MGC803 and AGS, were gotten from the Cell Bank of the Chinese Academy of Science (Shanghai, China) or the American Type Culture Collection (ATCC, Manassas, VA, USA). ALL these cells were maintained in DMEM (Gibco BRL, Rockville, MD, USA) including 10% fetal bovine serum, and 1% penicillin/ streptomycin (Solarbio, Beijing, China) in a 37° C humidified chamber containing 5% CO_2_ [30, 31].

### Western blot assay

Attached please find Supplementary Materials.

### Immunocytochemistry (IHC)

GC surgically removed from 2019.3 to 2019.5 were chosen from the Department of Surgery of Nanfang Hospital, Southern Medical University. The experimental protocols were approved by The Ethics Committee of the Southern Medical University, China. Immunohistochemistry was performed as previously described [20, 30].

### Constructs and establishment of stable transfectants

Normal human complementary DNA (cDNA) corresponding to full-length HMGA1 (HMGA1 variant 1) was acquired by RT-PCR. Then the products of PCR were subcloned into the vector pENTER-FLAG (ViGene Biosciences, Rockville, MD, USA). The AGS and BGC-823 lines were transfectants with pENTER vector and pENTER HMGA1vector to construct the stable cells line [30, 31].

### Transfection

Attached please find Supplementary Materials.

### Promoter reporter and dual luciferase assay

A 1.4-kb SUZ12 or CCDC43 promoter was cloned into pGL3-Basic Luciferase Reporter Vectors (Promega, USA). Empty pGL3-Basic vector was served as a negative control. QuikChange Site-Directed Mutagenesis kit (Stratagene, La Jolla, CA, USA) was applied to generate SUM12-MUT and CCDC43- MUT reporters. The mutation was confirmed by DNA sequencing. The cells were transfected with recombinant plasmids by using Lipofectamine 3000. The pRL-CMV vector (Promega) was served to standardize the transfection efficiency during all transfections. At 36-48 hours after transfection, we collected the cells and incubated with the reporter lysis buffer (Promega). The luciferase activity in cells were tested by a dual luciferase assay kit (Promega) following the instruction of manufacture. Promoter transcription activity was shown as the fold induction of relative luciferase unit (RLU) compared with basic pGL3 vector control. The sequences of oligonucleotide primers applied in this study is outlined in Supplementary Table 1.

### Chromatin immunoprecipitation (ChIP)

ChIP was carried out following the manufacturer’s instructions (ChIP Assay Kit, Upstate, USA). Briefly, cancer cells were collected and cross-linked with 1% formaldehyde, then cells were incubated for 10 min at 20° C. After stopping the reaction by glycine, the cells were lysed by SDS lysis buffer supplied with protease inhibitor, then sonicated to produce chromatin fragments between 200 to 500 bp. Following centrifugation, using ChIP dilution buffer diluted the clear supernatant 10-fold and incubated at 4° C overnight with an anti-HMGA1 (Abcam, ab4078, 1:200, Cambridge, UK). The protein-DNA complex was purified and DNA was extracted by phenol- chloroform, subsided with ethanol. Immunoprecipitates containing IgG antibody served as controls. PCR products were observed on a 2% agarose gel. The ChIP primers are listed in Supplementary Table 1.

### 5-Ethynyl-2′-deoxyuridine (EdU) assay

Attached please find Supplementary Materials.

### Plate colony formation assay

Attached please find Supplementary Materials.

### Wound healing assay (migration assay)

Attached please find Supplementary Materials.

### Invasion assays (Transwell assay)

Attached please find Supplementary Materials.

### Construction and production of recombinant lentivirus

Attached please find Supplementary Materials.

### *In vivo* experimental metastasis mouse models

Attached please find Supplementary Materials.

### Statistical analysis

All data were presented as the means ± standard deviation (SD), and all the experiments were repeated three times. Statistical analysis was applied by using SPSS Statistical software version 20.0 (IBM, Chicago, IL, USA). Survival curves were plotted by Kaplan-Meier analysis and compared with log-rank tests. Differences were analyzed by two-tailed Student's *t*-test. Values of *p* ≤ 0.05 were considered statistically significant.

## RESULTS

### HMGA1 expression is associated with the malignant biological behavior of GC

**Figure 1:**
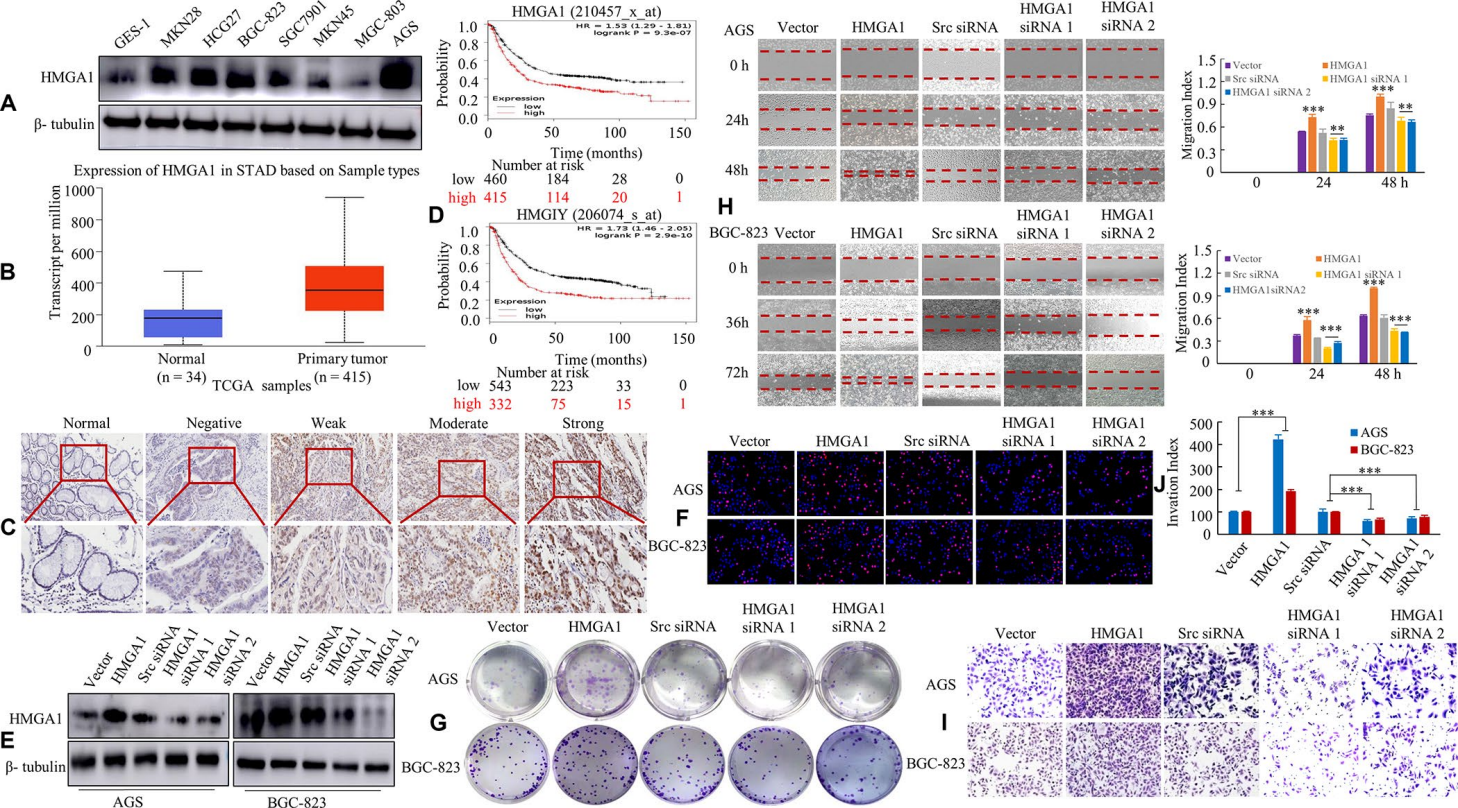
**HMGA1 expression is associated with the biological behavior of GC.** (**A**) HMGA1 protein levels were assessed in GC cell lines and a human normal gastric epithelial cell line *GES-1* using western blotting. β- tubulin was used as the internal control. (**B**) The protein expression of HMGA1 in GC and normal tissues analyzed by UALCAN cancer database. (**C**) IHC signal intensities were scored as nontumorous gastric mucosa (Normal), negative, weak, moderate and strong expression of HMGA1 protein in GC tissue. (**D**) OS survival curves of all GC patients cohorts (N = 875 and N = 875) from the KM plotter databases. N = number; OS, overall survival. (**E**) The protein levels of HMGA1 in AGS and BGC-823 cells with three treatments [Scrambled (Scr) siRNA, HMGA1 siRNA 1 and HMGA1 siRNA 2] determined by western blot analysis. (**F**) The GC cells transfected with the ectopic expression or knockdown of HMGA1 gene at 48 h and then stained with EdU and Hoechst 33342. (**G**) The AGS and BGC-823 cells were tested for the ability to form soft agar colonies. (**H**) Relative wound density at different time points of GC cells over a period of 48 h or 72 h. The measurements are from wounds made on a monolayer of GC cells cultured in the presence of different coating treatments and control. Original magnification, 10x. **, P < 0.05 and ***, P < 0.01. (**I**, **J**) *In vitro* the invasive ability of AGS and BGC-823 *cells* were evaluated by Transwell assay. The relative ratio of invasive cells was counted. ***, P < 0.01. Scale bars, 50 μm in (**C**); 100 μm in (**F**).

We first determined the level of HMGA1 in normal human gastric epithelial cells (GES-1) and seven different GC cell lines (MKN28, HCG27, BGC-823, SGC7901, MKN45, MGC803 and AGS). As expected, HMGA1 was highly expressed in 6 GC cells lines, except MGC803 cells, compared with GES-1 cells (Figure 1A). The analysis with ualcan database showed the higher protein expression of HMGA1 was also detected in GC tissues (Figure 1B). Secondly, we investigated the association between high expression of HMGA1 gene and the clinicopathological features of GC based on the IHC data of 51 GC samples. The results were shown in Supplementary Table 2, and Figure 1C shows a representative picture of gastric tissues. Increased HMGA1 expressional levels is closely related with differentiation (P= 0.028), lymph node metastasis (P= 0.004), tumor size (<5 cm3 vs ≥ 5 cm3, P = 0.009), AJCC stage (T1/T2 vs. T3/T4, P = 0.035) and TNM stage (I/II vs. III/IV, P= 0.002). Nevertheless, no significant correlation was found between HMGA1 expression and age (<60 y vs ≥60 y, P = 0.249) or sex (P = 0.764). The Kaplan-Meier (KM) curves obtained from the KMplot database (http://www.kmplot.com/gastric) demonstrated that high HMGA1 expression significantly reduced the overall survival of patients with GC (Figure 1D).

We transfected with HMGA1, vector plasmid, HMGA1 siRNA 1 or 2 or scrambled siRNA (src siRNA) in AGS and BGC-823 cells and confirmed transfection by western blot analysis (Figure 1E). We thirdly determined whether HMGA1 promotes cell proliferation in GC using both the EdU and soft agar assays. As expected, the overexpression of HMGA1 promoted cell proliferation, while silencing HMGA1 repressed cell proliferation in AGS and BGC-823 cells (Figure 1F, 1G and Supplementary Figure 1A, 1B).

Wound-healing and transwell assays were applied to assess the role of HMGA1 in cell migration and invasion. Compared to empty vector group, the stable transfectants of HMGA1 GC group showed notably increased migration, while *siRNA*-mediated knockdown of HMGA1 dramatically blocked the migratory ability of GC cells (Figure 1H). Fourthly, the invasiveness of AGS and BGC-823 cells after HMGA1 overexpression was elevated compared with that of the control group, whereas HMGA1 silencing weakened the invasive capabilities of cells (Figure 1I, 1J).

These results verified that HMGA1 expression is positively related with the malignant biological behavior of GC.

### Direct transcriptional mediation of SUZ12 or CCDC43 levels by HMGA1 in GC cells

**Figure 2:**
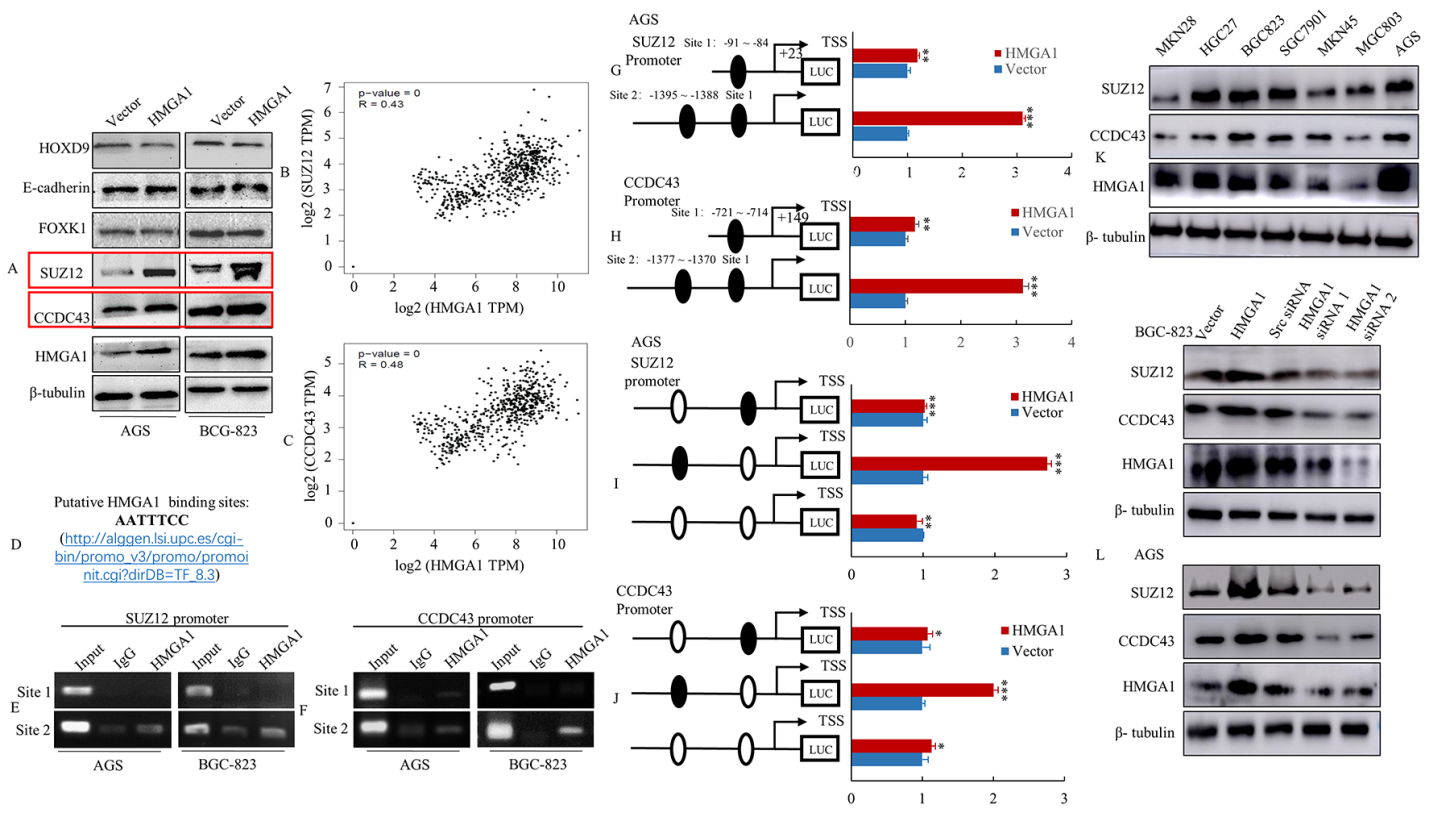
**HMGA1 directly binds to the SUZ12 or CCDC43 promoter and up-regulated the SUZ12 or CCDC43 promoter activity.** (**A**) The vector and HMGA1 plasmid were transfected into AGS and BGC-823 cells. The HOXD9, E-cadherin, FOXK1, SUZ12, CCDC43 and HMGA1 expression levels were detected in AGS and BGC-823 cell lines by western blot assay. (**B**, **C**) The relationship between HMGA1 and SUZ12 or CCDC43 levels was analyzed by *GEPIA* bioinformatics tool. (**D**) List of consensus HMGA1 sequence. (**E**, **F**) Binding of HMGA1 to the SUZ12 or CCDC43 promoter *in vivo*. ChIP assays were done with AGS and BGC-823 cells treated with anti-HMGA1 or IgG. (**G**, **H**) HMGA1 transactivates SUZ12 or CCDC43 promoter activities in AGS cells. The SUZ12 or CCDC43 promoter construct was cotransfected with HMGA1 or vector, and the relative luciferase activity was determined. **, P < 0.05; ***, P < 0.01. (**I**, **J**) Selective mutation analyses identified HMGA1-responsive regions in the SUZ12 or CCDC43 promoter. Mutated SUZ12 or CCDC43 promoter constructs were cotransfected with HMGA1 and relative luciferase activities were determined**.** *, P > 0.05; **, P < 0.05; ***, P < 0.01. (**K**) Western blot assay were used to detect the expression of SUZ12, CCDC43 and HMGA1. (**L**) The protein levels of SUZ12 and CCDC43 in AGS and BGC-823 cells with three treatments [Scrambled (Scr) siRNA, HMGA1 siRNA 1 and HMGA1 siRNA 2] determined by western blot analysis.

Our previously studies indicated that genes including SUZ12 [20], E-cadherin [20], FOXK1 [29], CCDC43 [29] and HOXD9 [31], are implicated in the pathogenesis of GC. As a transcription factor, several downstream genes may be regulated by HMGA1; therefore, we further assessed whether the ectopic expression of HMGA1 regulated the expressional levels of a group of genes (SUZ12, E-cadherin, FOXK1, CCDC43 and HOXD9) in GC cells. The results indicated that increased HMGA1 expression significantly upregulated the levels of SUZ12 and CCDC43, while HOXD9, E-cadherin and FOXK1 protein levels remained unchanged upon HMGA1 overexpression (Figure 2A). Then, GEPIA databases were used to evaluate the relationship between HMGA1 and SUZ12 or HMGA1 and CCDC43 to identify co-expression genes. As a result, a strikingly positive relationship between HMGA1 and SUZ12 or HMGA1 and CCDC43 was established in databases (*R* = 0.43 and R = 0.48, Figure 2B, 2C).

To determine whether SUZ12 or CCDC43 could be direct transcriptional targets of HMGA1, we first checked approximately 1400 bp of the promoter region of SUZ12 or CCDC43 accompany with HMGA1 DNA-binding consensus sequence (Figure 2D). The SUZ12 gene promoter contains two putative HMGA1 binding sites (Site 1: -91 to -84 and Site 2: -1395 to -1388), and the CCDC43 gene promoter region includes two HMGA1 putative binding sites (Site 1: -721 to -714 and Site 2: -1377 to -1370). To further investigate whether HMGA1 binds directly to the human SUZ12 or CCDC43 promoter, ChIP assay was applied. As expected, with using antibodies specific to HMGA1, ChIP assays in AGS and BGC-823 cells indicated the promoter of endogenous SUZ12 or CCDC43 can be bound directly with HMGA1 protein (Figure 2E, 2F).

We cloned the promoter regions of HMGA1 site 1 (HMGA1p1) and HMGA site 2 (HMGA1p2) of human SUZ12 or CCDC43 upstream of a luciferase gene in a reporter plasmid. To investigating whether the SUZ12 or CCDC43 promoter was activated via upregulating of HMGA1, the transient transfection was applied. Dual-luciferase assays suggested that compared with empty vector group, the activity of HMGA1p2 in SUZ12 cells enhanced 3.1 ~ 3.4-fold and CCDC43 cells increased 3.1 ~ 3.2-fold, while the magnification shown a mild declination with HMGA1p1 transfection in SUZ12 or CCDC43 cells (Figure 2G, 2H and Supplementary Figure 2A, 2C).

Promoter mutation assays were further applied to verified the results. Various mutant reporters were obtained from the wild-type SUZ12 or CCDC43 promoter construct, including a HMGA1-binding site 1 mutation only (SUZ12-Mut 1 or CCDC43-Mut 1), a HMGA1-binding site 2 mutation only (SUZ12-Mut 2 or CCDC43-Mut 2), and a mutation of both sites 1 and 2 (SUZ12-Mut 3 or SUZ12-Mut 3). These mutant luciferase reporters were transfected into AGS or BGG-823 cells, then their activity was compared with that of the wild-type SUZ12 and CCDC43 promoters. Disruption of the HMGA1-binding site 2 significantly attenuated SUZ12 or CCDC43 promoter activity in AGS and BGC-823 cells (Figure 2I, 2J and Supplementary Figure 2B, 2D).

To verify the relationship between SUZ12 and HMGA1 or CCDC43 and HMGA1, the expression of SUZ12, CCDC43 and HMGA1 was tested in GC cell lines. The data indicated the levels of protein in SUZ12 and HMGA1 or CCDC43 and HMGA1 were positively link in the majority of these GC cell lines (Figure 2K). Further, overexpression of HMGA1 up-regulated SUZ12 and CCDC43 expression, whereas the knockdown of HMGA1 decreased SUZ12 and CCDC43 levels in BGC-823 and AGS cells (Figure 2L). These data implied that SUZ12 and CCDC43 are direct transcriptional targets of HGMA1.

### SUZ12 is essential for HMGA1-mediated GC growth and metastasis

**Figure 3:**
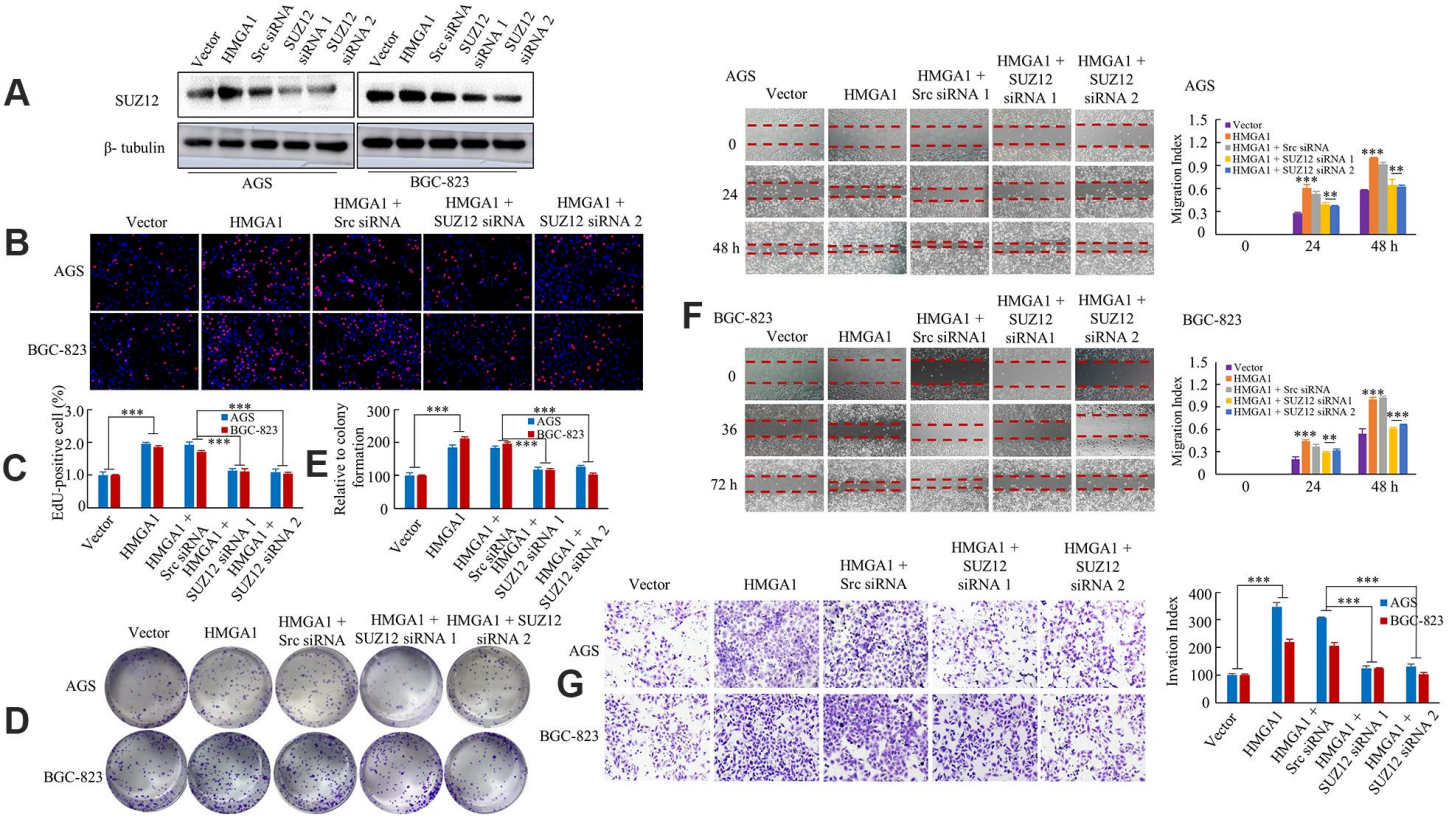
**HMGA1 -SUZ12 axis promote development and progression.** (**A**) Western blot analysis of SUZ12 expression in AGS and BGC-823 cell lines. (**B**, **C**) The AGS and BGC-823 cells, cultured for 48 hours following transfection with vector, HMGA1, HMGA1 + Src siRNA, HMGA1 + SUZ12-siRNA 1 or HMGA1 + SUZ12-siRNA 2, were subjected to the EdU incorporation assay. ***, P < 0.01. (**D**, **E**) The colony-forming cell assay was performed of *GC cells*. ***, P < 0.01. (**F**) The monolayers of AGS and BGC-823 cells were scratched wounded in a one-direction pattern. **, P < 0.05 and ***, P < 0.01. (**G**) Transwell assays were employed to determine the invasion ability of control and transfected GC cells. ***, P < 0.01. Scale bars, 100 μm in (**B**).

Next, we determined whether SUZ12 take part in HMGA1-mediated proliferation and metastasis. SUZ12 was downregulated by using siRNA in HMGA1-overexpressing cells, and the effect was verified by western blot (Figure 3A). We observed that the overexpression of HMGA1 enhanced the capability of GC cell proliferation by EdU incorporation and colony formation assays. On the contrary, downregulation of SUZ12 decreased HMGA1-mediated proliferation of AGS and BGC-823 cells (Figure 3B–3E). Furthermore, the up-regulation of HMGA1 significantly enhanced the migration and invasion of GC cell, while the down-regulation of SUZ12 aborted the declined migration and invasion abilities stimulated by HMGA1 overexpression (Figure 3F, 3G). Therefore, SUZ12 is necessary for HMGA1-mediated GC growth and metastasis.

### CCDC43 is essential for HMGA1-mediated GC growth and metastasis

**Figure 4:**
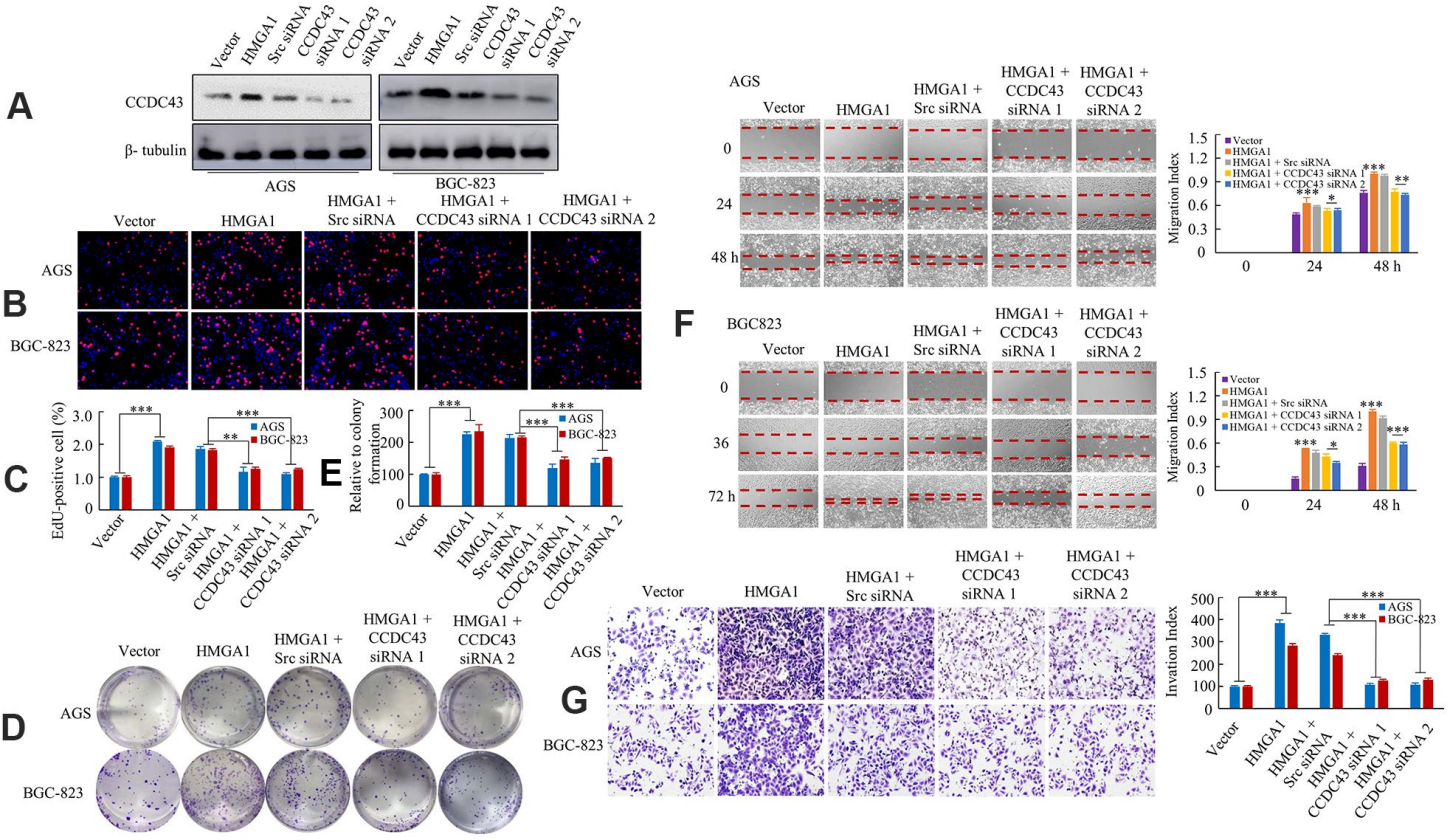
**HMGA1 -CCDC43 axis promote development and progression.** (**A**) Western blot analysis of CCDC43 expression in AGS and BGC-823 cell lines. (**B**, **C**) DNA synthesis of AGS or BGC-823 cells were measured by EdU incorporation assay after the indicated transfection. **, P < 0.05; ***, P < 0.01. (**D**, **E**) The growth of GC cells were examined with colony forming assay. ***, P < 0.01. (**F**) The GC cell migration assays were performed. *, P > 0.05; **, P < 0.05; ***, P < 0.01. (**G**) Transwell assays were performed to determine the invasion capacity of control and transfected GC cells. ***, P < 0.01, 100 μm in (**B**).

Similarly, we treated the AGS and BGC-823 cells expressing CCDC43 in HMGA1-overexpressing cells with CCDC43 siRNA 1 and 2. As expected, compared with control siRNA, CCDC43-siRNA decreased the CCDC43 expression by western blot (Figure 4A). We observed that HMGA1 overexpression promoted GC proliferation, while knockdown of CCDC43 was exhibited a negative effect on HMGA1-induced DNA synthesis using an EdU incorporation assay (Figure 4B, 4C). The ectopic expression of HMGA1 increased GC cell growth, whereas CCDC43 downregulation decreased the HMGA1-mediated proliferation of AGS and BGC-823 cells, as shown using a colony formation assay (Figure 4D, 4E).

The functional roles of HMGA1 and CCDC43 in GC cell migration and invasion were further tested. Up-regulation of HMGA1 enhanced GC cell migrative ability and invasion capacity. In contrast, CCDC43 silencing in HMGA1-overexpressing cells cause a declination in the migratory and invasion potentials of HMGA-upregulation cells (Figure 4F, 4G).

Taken together, these data showed that HMGA1 promotes GC growth and metastasis by transactivating the expressional levels of CCDC43.

### Identification of an association of HMGA1, SUZ12 and CCDC43 in human GC

**Figure 5:**
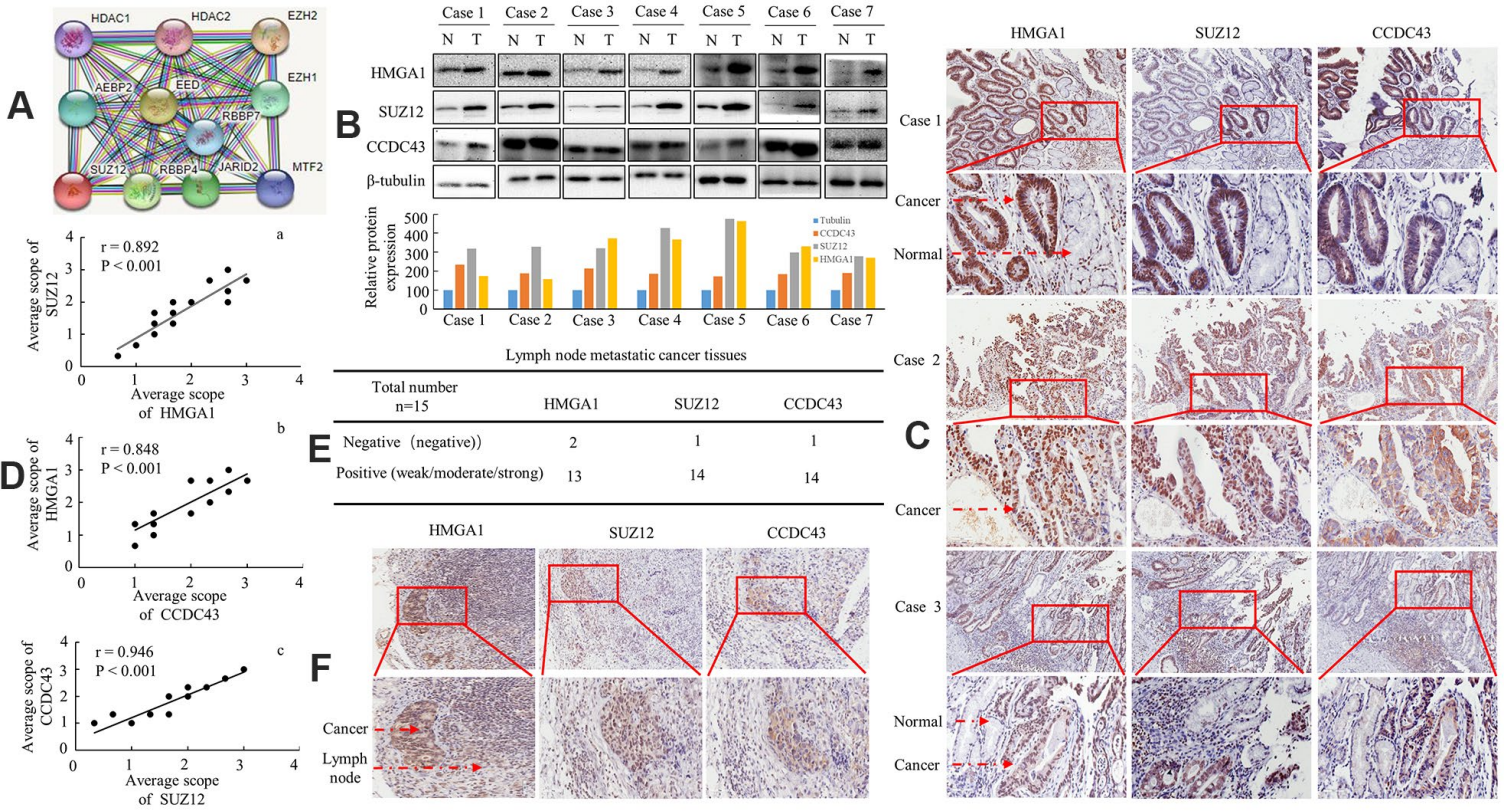
**Protein expression level of HMGA1, SUZ12 and CCDC43 in human GC patients.** (**A**) SUZ12-related protein-protein interaction (PPI) network from the STRING database. (**B**) Expression of HMGA1, SUZ12 and CCDC43 protein in each of the primary GC (T) and adjacent noncancerous tissues (N) paired from the same patient by western blotting. The protein expression levels were quantified by comparing the gray level of each band using Quantity One Software (below). (**C**) Immunohistochemical staining of HMGA1, SUZ12 and CCDC43 in gastric tissues. (**D**) The correlation between SUZ12 and HMGA1, between CCDC43 and HMGA1, or between SUZ12 and CCDC43 in GC tissues. (**E**, **F**) Immunohistochemical analysis of three protein expression in metastatic lymph nodes. Scale bars, 50 μm in (**C**, **F**).

We observed no interaction between SUZ12 and CCDC43 proteins using the STRING database (Figure 5A). We further investigated HMGA1, SUZ12 and CCDC43 expression in clinical specimens. The three genes were highly upregulation in the seven examined tumor samples paired with adjacent non-neoplastic mucosal tissues by western blot (Figure 5B). IHC staining revealed that these three genes were also strongly or moderately expressed in all nineteen primary GC tissue samples, whereas the three proteins did not expressed, or weakly expressed in adjacent non-neoplastic tissues, as shown in Figure 5C. Positive correlations were found between HMGA1 and SUZ12 (Figure 5D-a), between HMGA1 and CCDC43 (Figure 5D-b) and between CCDC43 and SUZ12 (Figure 5D-c) in the nineteen GC tissues by linear correlation analyses.

Then, the expressional levels of three genes in regional lymph nodes related with metastasis were tested as well. In total, 13/15, 14/15 and 14/15 of the metastatic tissues taken from lymph nodes highly expressed HMGA1, SUZ12 and CCDC43 by means of IHC, as exemplified in one patient (Figure 5E, 5F). Thus, overexpression of HMGA1, SUZ12 and CCDC43 is associated with enhanced regional lymph node metastasis in human GC.

Together, these results implying that the HMGA1-SUZ12/CCDC43 signal axis might be an attractive target for GC therapeutic interventions.

### SUZ12/CCDC43 is necessary for HMGA1-induced GC metastasis *in vivo*

**Figure 6:**
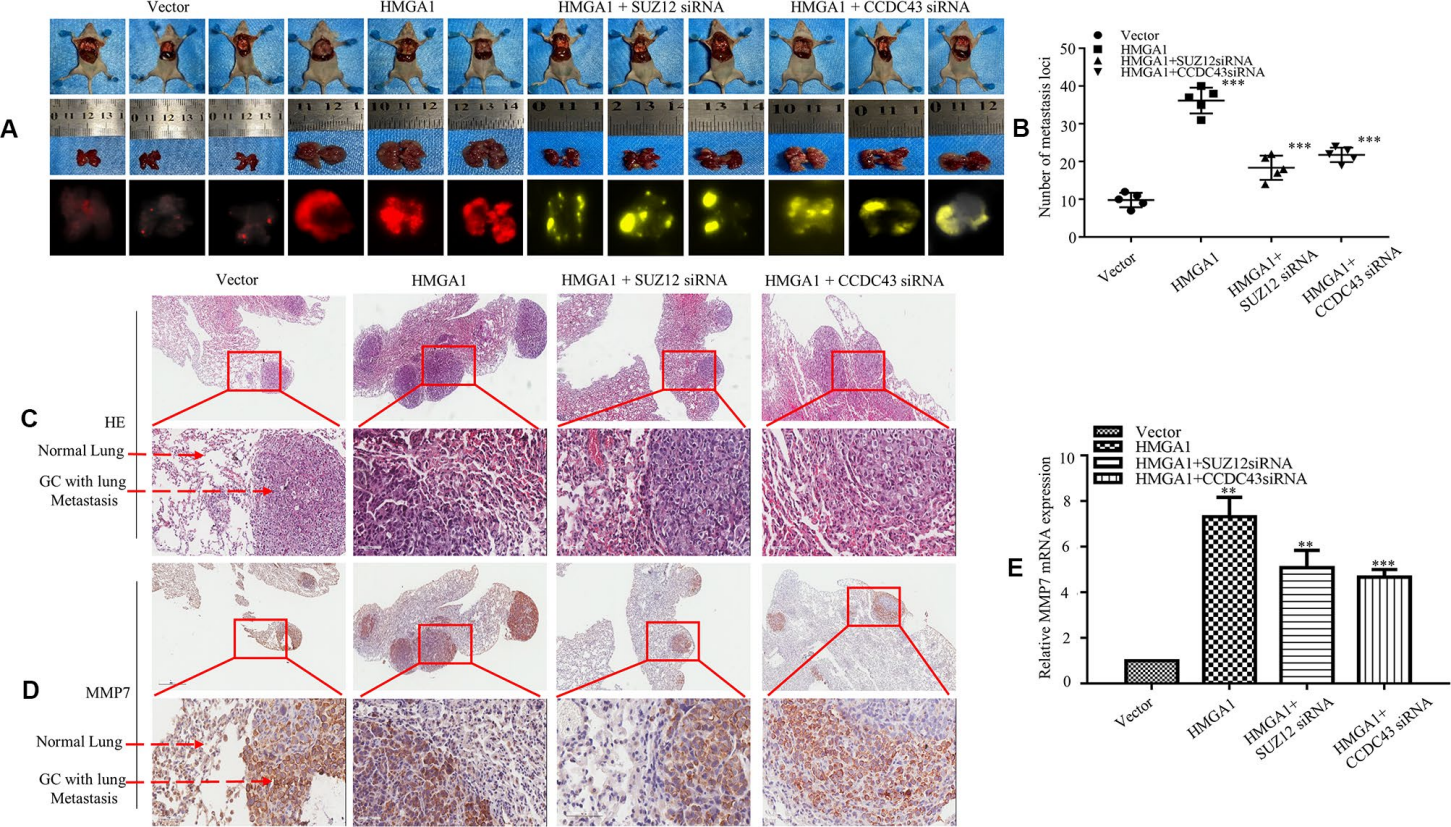
**SUZ12/CCDC43 is necessary for HMGA1-induced GC metastasis *in vivo*.** (**A**) Whole-body fluorescence imaging of GC progression in mice *(n = 3)*. Images of metastatic loci in the lungs by arrows. (**B**) Number of metastatic loci in lung was counted. ***, P < 0.01. (**C**) H&E staining of lungs was performed in samples from mice. (**D**) MMP7 expression in the lung metastasis of GC was detected by IHC. (**E**) Expression of MMP7 in lung tumours derived from AGS cells was determined by qRT-PCR. **, P < 0.05; ***, P < 0.01. Scale bars, 50 μm in (**C**, **D**).

Next, a tail vein metastatic assay in nude mice was carried out to investigate the metastatic capacity of AGS cells *in vivo*, and organs were scanned for metastasis by a visualization system as well. We showed that metastatic lesions were grown in the lungs of mice (Figure 6A). Compared with the LV-vector group, more large lung metastatic nodules were found in LV-HMGA1 groups. In contrast, LV-HMGA1-SUZ12-shRNA1 or LV-HMGA1-CCDC43-shRNA1 cells reversed the effects observed in LV-HMGA1 cells (Figure 6B). H&E staining assays confirmed a metastasis in the lung (Figure 6C). Moreover, the IHC assay showed that MMP7 protein expression was enhanced in cancer tissues compared with adjacent normal lung (Figure 6D).

The qPCR assay show upregulation of HGMA1 leading to a highly increase of MMP7 protein, whereas downregulation of SUZ12 or CCDC43 in HMGA1-overexpressing cells resulted in a decrease in MMP7 protein (Figure 6E). These results indicate that SUZ12/CCDC43 is necessary for HMGA1-induced GC metastasis *in vivo*.

## DISCUSSION

In this work, we observed the level of HMGA1 was markedly enhanced in human GC cells and related with an unfavourable prognosis. Moreover, the overexpression of *HMGA1* enhanced cell growth and metastasis of AGS and BGC-823 cells *in vitro*. Mechanistic studies revealed that HMGA1 bound to the promoter of SUZ12 or CCDC43 and promoted the expression of SUZ12 or CCDC43. Therefore, HMGA1 stimulates GC proliferation and metastasis via transactivating SUZ12 and CCDC43 expression.

Growing evidence indicates that HMGA1 is an important oncoprotein [32, 33] and ectopic expression of HMGA1 is correlated with unfavorable outcomes of patients in cancer [34, 35]. Its overexpression in cancer is largely due to transcriptional, posttranscriptional and post-translational mechanisms. For example, the transcriptional activation of the *fra-1* and TCF-4 gene upregulates HMGA1 mRNA expressional level [36, 37]. Moreover, HMGA1 mRNA is the target of different miRNAs, the frequent downregulation of which cause HMGA1 mRNA overaccumulation [38, 39]. In addition, upregulation of HMGA1 pseudogenes can interpret the advancement of the expression of HMGA1 by decoying the miRNAs targeting its mRNA as well [40]. Besides, HMGA1 is modulated through its post-translational protein modifications such as methylation, acetylation and phosphorylation in cancer [41, 42]. Nevertheless, the molecular mechanisms underlying HMGA1 regulation in GC have not been entirely elucidated. In this study, we revealed that most GC cell lines expressed high levels of HMGA1. Additionally, HMGA1 might function as a candidate unfavorable prognostic marker for human GC by bioinformatics analysis. Some studies have shown that overexpression of HMGA1 improves the proliferation and migration/invasion abilities of cells [43, 44]. Consistently, we demonstrated that HMGA1 facilitated the growth and invasion of AGS and BGC-823 cells by gain-of-function and loss-of-function experiments. Thus, our observations indicate that the forced expression of HMGA1 might have a function in the onset and development of GC.

HMGA1 acts as a transcription factor and can stimulate or suppress the activity of genes by binding to their control regions. We examined the number of promoters which contain potential HMGA1-binding sites. We revealed that overexpression of HMGA1 notably increased the levels of SUZ12 and CCDC43. Thus, SUZ12 and CCDC43 may be transcriptional targets of HMGA1. SUZ12 (polycomb protein SUZ12) is a zinc finger gene encoding zeste homolog 12 protein. SUZ12 protein, EZH2 and EED, also forms various Polycomb repressive complexes [45]. SUZ12 is a crucial regulator of multiple cellular functions and is transcriptionally regulated by transcription factor genes [46]. Some studies have reported that the transcription of NF-kappa B target genes was positively mediates by the interaction between EZH1-SUZ12 complex and UXT [47]. Overexpression of SUZ12 has been found to be a vital factor in GC cell proliferation and metastasis via regulating the expression of EMT and KLF2 [15]. Moreover, the expressional levels of FOXC1 gene is negatively related with that of Polycomb group (PcG) genes, i.e., Bmi1, EZH2, and SUZ12, in breast cancer cells [48]. Another CCDC43 gene functions as an oncogene in *GC*. CCDC43 encodes a member of the CCDC family and is involved in multiple aspects of gastrointestinal cancer, such as tumorigenesis, growth, invasion and metastasis [29, 30]. We previously showed that ectopic levels of CCDC43 might be a regulator or a trigger of epithelial-mesenchymal transition (EMT) in CRC cells. Moreover, promoter assays illustrated that promoter of human CCDC43 gene was directly bound and subsequently activated by FOXK1. [29]. Nevertheless, the underling mechanism of which HMGA1 regulates SUZ12 or CCDC43 expressional levels by transcriptional activation to stimulate cell proliferation, invasion and metastasis in GC remains unclear.

In this work, we confirmed our above findings that SUZ12 and CCDC43 are direct transcriptional targets of HMGA1, which is similar with some studies showing that HMGA1 transcriptionally regulates KIT ligand in breast and ovarian cancer cells [36], and HMGA1 and HMGA2 proteins positively regulate Pit1 promoter activity in pituitary adenoma *GH3* and αT3 cell lines [49]. The conclusion is based on the following observations. First, SUZ12 or CCDC43 promoter activity was significantly higher in AGS and BGC-823 cells overexpressing HMGA1. Second, ChIP and luciferase assays indicated that HMGA1 protein binds to AT-rich regions of SUZ12 or CCDC43 promoter DNA *in vitro*. Third, mutations of the AT-rich regions caused blockage of HMGA1 transcriptional activity. Four, SUZ12 or CCDC43 silencing repressed growth and metastatic potential stimulated via upregulation of HMGA1 in GC *in vivo*. However, the possible mechanisms and detailed interplay among them need further investigation.

Some studies have found HMGA1, SUZ12 and CCDC43 were involved in the clinical significance in a great deal of tumors. Abe et al. expounded that the expression of HMGA1 protein were notably higher in cancerous tissues than non-cancerous tissues and that higher HMGA1 expression was positively related with lymph node metastasis and advanced clinical stage in breast cancer [9]. Xia et al. proved that the levels of SUZ12 was remarkably advanced in 64 GC tissues compared to normal tissues [15]. Moreover, aberrant overexpression of SUZ12 was significantly associated with aggressive clinicopathological features and inferior survival [50]. Our study have implied that CCDC43 as an oncogenic factor in gastrointestinal cancers [29, 30]. Additionally, increased the levels of CCDC43 have a close relationship with clinicopathological features and unfavorable prognosis in GC [30]. In present work, we found that the levels of HMGA1 was positively related with SUZ12 or CCDC43 expression in tumor samples by Spearman’s correlation. Furthermore, HMGA1, SUZ12 or CCDC43 expression was related with lymphatic metastasis in GC patients. These studies indicate that HMGA1 induces SUZ12 or CCDC43, potentially contributing to GC growth and metastasis.

Taken together, this study provides convincing evidence that HMGA1 has an basic role in GC cell proliferation and metastasis by regulating the proto-oncogene SUZ12 or CCDC43. Thus, HMGA1 might become a potential favorable target for prevention of GC cell proliferation and metastasis.

## Supplementary Material

Supplementary Materials and Methods

Supplementary Figures

Supplementary Tables
