# Supplementary material for: HMGA1 promotes gastric cancer growth and metastasis by transactivating SUZ12 and CCDC43 expression
Source: Aging (Albany NY). 2021 Jun 24;13(12):16043–61. doi: 10.18632/aging.203130 (PMC8266323; doi:10.18632/aging.203130)
Supplement: Supplementary Materials and Methods [file aging-13-203130-s001.pdf]

SUPPLEMENTARY MATERIALS AND METHODS

Antibodies

Rabbit anti-HMGA1 antibody (ab129153) and (sc - 393213) were purchased from Abcam (Abcam, Cambridge, United Kingdom) and Santa Cruz (Santa Cruz, California, USA), respectively. Rabbit anti-SUZ12 antibody (ab12073) and (#3737) were purchased from Abcam (Abcam, Cambridge, UK) and Cell Signaling Technology (Cell Signaling Technology, Massachusetts, USA), respectively. Rabbit anti-CCDC43 antibody (NBP1-83536) was purchased from Novus Biologicals (Novus Biologicals, Colorado, USA). Rabbit anti- MMP7 antibody (#3801) was purchased from Cell Signaling Technology (Cell Signaling Technology, Massachusetts, USA). Mouse anti-β-tubulin (RM2003) was purchased from Beijing Ray Antibody Biotech (Beijing Ray Antibody Biotech, Beijing, China). Rabbit anti-E-cadherin (20874-1-AP) was acquired from Proteintech (Wuhan, China). Rabbit anti-HOXD9 antibody (ab90260) and anti-SP1(ab13370) were acquired from Abcam (Abcam, Cambridge, UK). Mouse anti-FOXK1 antibody (sc-373810) was purchased from Santa Cruz (Santa Cruz, California, USA).

Western blot assay

Cellular protein extracts were homogenized in an ice-cold RIPA lysis buffer (Beyotime Institute of Biotechnology, Beijing, China) for 30 min. The lysates were centrifuged at 4° C, and protein concentrations were determined using an Enhanced BCA Protein Assay Kit (Beyotime Institute of Biotechnology, Beijing, China). Thirty µg of each protein sample was separated by 10% sodium dodecyl sulfate polyacrylamide gel electrophoresis and electro-transferred onto a polyvinylidene difluoride membrane. After blocking with nonfat milk, the membranes were incubated with the primary antibodies at 4° C overnight. The membranes were then washed with Tris-Buffered Saline and Tween 20 followed by incubation with HRP-conjugated secondary antibodies (1:5000) at room temperature. Immunoreactivity was visualized by ECL chemiluminescence (FDbio Science, China). The relative protein expression levels were quantified by comparing the gray level of each band using Quantity One Software (Life Science Research, Hercules, CA, USA).

Immunocytochemistry (IHC)

Nineteen GC surgically removed from 2019.3 to 2019.5 were selected from the Department of Surgery of Nanfang Hospital, Southern Medical University. The Ethics Committee of the Southern Medical University, China, approved the experimental protocols. Paraffin-embedded specimens were cut into 4-µm sections and baked at 65° C for 30 min. The slides were deparaffinized with xylene, rehydrated with ethanol, washed and subjected to microwave antigen retrieval in a citrate buffer. Sections were then immersed in 3% hydrogen peroxide to block endogenous peroxidase activity and were incubated with the primary antibodies followed by incubation with peroxidase-conjugated anti-rabbit secondary antibody (Dako) (1:100). The expression of HMGA1, SUZ12 or CCDC43 was then visualized using 1 mg/ml 3, 3#-diaminobenzidine and counterstained with hematoxylin. Normal mouse IgG (Sigma) was used as an isotype control for anti-HMGA1, SUZ12 or CCDC43 antibody to verify specificity of the staining. The results were independently scored by two observers. The staining results were classified according to the carcinoma cell staining intensity as follows: 0, negative staining; 1, weak staining; 2, moderate staining; and 3, strongly staining. These two scores were multiplied to obtain the final scores, which were used for statistical analysis. We defined negative- and weak-stained cells as low expressers, and cells that were moderate- and intense-stained were considered to be high expressers of this protein.

Transfection

The cells of 70-80 % confluency in six-well plates were transfected with HMGA1 expression plasmid (pENTER-FLAG - HMGA1) or control vector, or HMGA1-siRNA (The sequences of HMGA1-siRNA were: siRNA 1: CAACTCCAGGAAGGAAACCAA; siRNA 2: AGCGAAGTGCCAACACCTAAG) ;or SUZ12-siRNA (The sequences of SUZ12-siRNA were: siRNA 1: GTCTCATCGAAACTCCAGA; siRNA 2: CAGCCATATGGTGAAGTCT); or CCDC43-siRNA (The sequences of CCDC43-siRNA were: siRNA 1: CGTTTATGGAGCCTACATC; siRNA 2: AGCCTACATCTTGGGTATC), or negative control siRNA (NC) (RiboBio, Guangzhou, China) using Lipofectamine 3000 reagent (Invitrogen, Carlsbad, CA, USA) according to the manufacturer's instruction. The

negative siRNA sequences had been confirmed not to interact with any mRNA sequence.

5-Ethynyl-2′-deoxyuridine (EdU) assay

The EdU assay kit (RiboBio, China) was used to measure cell proliferation. The cells were cultured in 24-well plates (1×10^5 cells/well) with RPMI 1640 for 24 h. After transfection, cells were incubated with 5-ethynyl-2'-deoxyuridine (EdU, RiboBio, China) for 5 h before staining. The cell proliferation was detected using Cell-Light™ EdU Cell Proliferation Detection Kit (RiboBio, China) following the manufacturer's instructions. EdU positive cells were observed under a fluorescence microscope after Apollo staining and 4',6-diamidino-2-phenylindole (DAPI) staining (Thermo Fisher Scientific, Waltham, MA, USA).

Plate colony formation assay

After 24 h of transfection, GC cells were trypsinized, adjusted to single cell suspensions, plated into 60 mm dishes at 500 cells/well and cultured in RPMI 1640 medium for 12 days. Then, the dishes were washed with PBS, and the colonies were fixed with 4 % paraformaldehyde for 1 h and stained with 0.1 % crystal violet solution for 30 min. The number of colonies was counted. All the experiments were performed in triplicate.

Wound healing assay (migration assay)

The GC cells (5×10^5) were seeded in six-well plate with 100% confluence. Linear scratch wounds were created by 200 µl sterile pipette tip. Next, the plate was washed by PBS for several times to remove the suspended cell and the cells were cultured in serum-free media. After 0 and 48 or 72 h, the cells were cultured in the presence of 10 µg/ml mitomycin C to inhibit cell proliferation. Then, we imaged the wounds at the same position under the microscope and the distance between the wound sides was calculated. Experiments were performed in triplicate.

Invasive ability assays (Transwell assay)

The GC cell invasion was assessed using Matrigel invasion chamber (BD Biosciences, Franklin Lakes, NJ, USA), as per the protocol provided by the manufacture. Briefly, the transfections were resuspended in serum-free media. Then, 5×10^4 cells were placed in each Transwell membrane filter inserts, the lower chamber was filled with 600 µl of complete medium, and the samples were incubated for an additional 24 h. The invading cells on the underside of the membrane were fixed with 4 % paraformaldehyde for 1 h and then were stained in 0.1 % crystal violet. The numbers of invading cells were calculated using a microscope (Olympus IX51; Olympus, Tokyo, Japan) at a magnification of x200 in five different random fields. The mean of triplicate assays for each experimental condition was used.

Construction and production of recombinant lentivirus

Lentiviruses expressing HMGA1 (LV- HMGA1) were constructed by GeneChem (Shanghai, China) using the Ubi-MCS-3FLAG-CBh-Cherry-IRES-puromycin vector (absorption 587 nm, emission 610 nm). Ubi-MCS-3FLAG-CBh-Cherry-IRES-puromycin empty vectors were used as controls (Shanghai GeneChem Co., Ltd., China).

Double-stranded oligonucleotides encoding human SUZ12-vshRNA (NM_015355: SUZ12 shRNA 1: CCGGGTCTCATCGAAACTCCAGATCAAGAGTC TGGAGTTTCGATGAGACTTTTTG) or CCDC43-vshRNA (NM_144609: CCDC43 shRNA 1: CCGGCG TTTATGGAGCCTACATCTCAAGAGGATGTAGGC TCCATAAACGTTTTTG) were annealed and inserted into the U6-MCS-Ubiquitin-gcGFP-IRES-puromycin short hairpin RNA (shRNA) expression vector (absorption 475 nm, emission 505 nm). Selected pools of overexpression and knockdown cells were used for subsequent experiments.

***In vivo* experimental metastasis mouse models**

BALB/C nude (nu/nu) mice (6–8 weeks, Female, SPF degree, 20 ± 3 g) were purchased from the Laboratory Animal Center of Southern Medical University (Guangzhou, China). All procedures were approved by the Institutional Animal Care Committee. All efforts were made to minimize animal suffering, reduce the number of animals used and utilize possible alternatives to *in vivo* techniques. To evaluate the *in vivo* metastatic potential of cancer cells, female *BALB/C-nu-nu* nude mice were injected with 4×10^5 lentivirus-vector,lentivirus-HMGA1,lentivirus-HMGA1-SUZ12 shRNA and lentivirus-HMGA1-CCDC43 shRNA-transfected cells per mouse (n = 3 mice/group) through the tail vein (TV). Post-TV injection, body weight and the status of nude mice were monitored every 3 days. The animals were euthanized 4 weeks after the injection, their lungs were removed, and individual organs were removed and assessed using the *In Vivo* F Imaging System (Kodak). Tissues were harvested for the H&E analysis, and immunohistochemistry (IHC) staining and qRT-PCR were performed.
