# Supplementary material for: HMGA1 promotes gastric cancer growth and metastasis by transactivating SUZ12 and CCDC43 expression
Source: Aging (Albany NY). 2021 Jun 24;13(12):16043–61. doi: 10.18632/aging.203130 (PMC8266323; doi:10.18632/aging.203130)
Supplement: Supplementary Figures [file aging-13-203130-s002.pdf]

SUPPLEMENTARY FIGURES

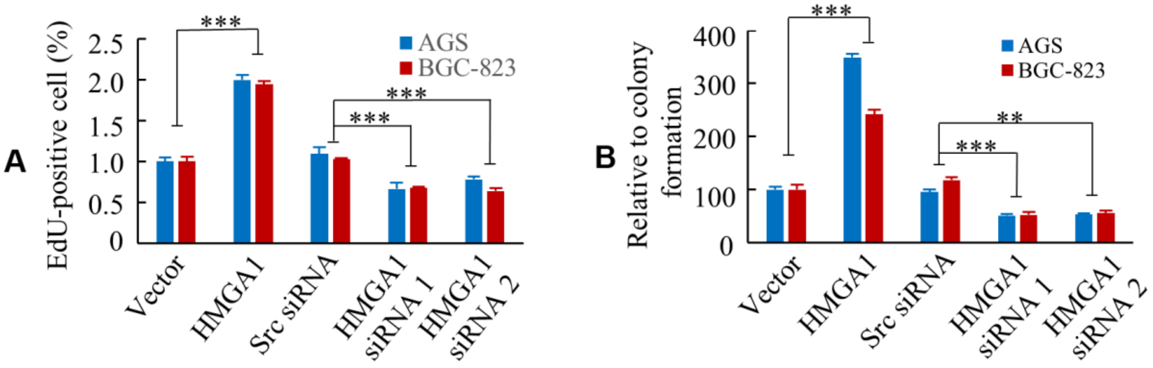

Supplementary Figure 1. Effect of HMGA1 on cell proliferation capacity. (**A**) The GC cells transfected with the ectopic expression or knockdown of HMGA1 gene at 48 h and then stained with EdU and Hoechst 33342. ***, P < 0.01. (**B**) The results are presented as the average number of colonies from three wells. **, P < 0.05 and ***, P < 0.01.

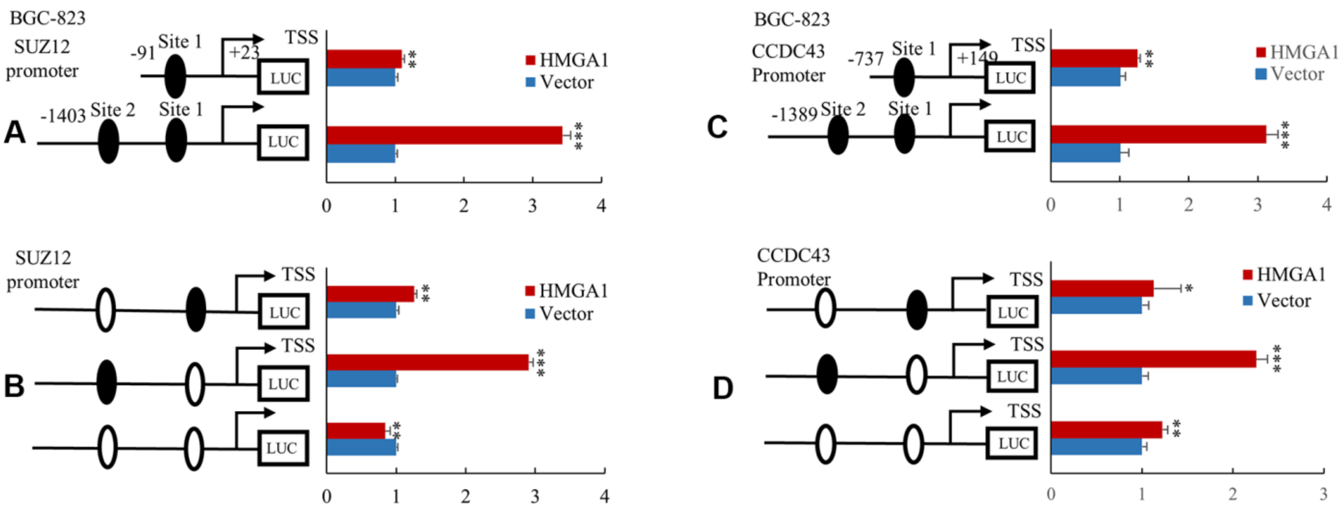

Supplementary Figure 2. HMGA1 transactivates SUZ12 or CCDC43 promoter activities. (**A**, **C**) The SUZ12 or CCDC43 promoter construct was cotransfected with HMGA1 or vector in BGC-823 cells, and the relative luciferase activity was determined. **, P < 0.05; ***, P < 0.01. (**B**, **D**) Selective mutation analyses identified HMGA1-responsive regions in the SUZ12 or CCDC43 promoter. Mutated SUZ12 or CCDC43 promoter constructs were cotransfected with HMGA1 and relative luciferase activities were determined. *, P > 0.05; **, P < 0.05; ***, P < 0.01.
