# Supplementary material for: HMGA1 promotes gastric cancer growth and metastasis by transactivating SUZ12 and CCDC43 expression
Source: Aging (Albany NY). 2021 Jun 24;13(12):16043–61. doi: 10.18632/aging.203130 (PMC8266323; doi:10.18632/aging.203130)
Supplement: Supplementary Tables [file aging-13-203130-s003.pdf]

SUPPLEMENTARY TABLES

Supplementary Table 1. Oligonucleotides sequences.

Experiment		Name	Position or orientation	Sequence (5’-3’)
Luciferase construction	SUZ12	R:	95~ 77	TCCGCTCGAG GATTCCCCCGTCAGTCAC(XhoI)
		L: SUZ12p1-WT	-91~ -84	F: 5’ - GGGGTACC***ATTTTCC***CGCGAATTCAGTT (Kpn I) -3’
		L: SUZ12p2-WT	-1395 ~ -1388	F: 5’- GGGGTACC AGGCTTCA***GGAAGTC***TGAGAA (Kpn I) -3’
	CCDC43	R:	271~ 251	TCCGCTCGAG CAAAGTCACGCGGCTAATAA (XhoI)
		L: CCDC43p1-WT	-737~ -714	F: 5’ - GGGGTACC TTGTTTGTTTTGAGAC***GGAATTT*** (Kpn I) -3’
		L: CCDC43p2-WT	-1389 ~ -1369	F: 5’- GGGGTACC GAAGCCTTCCTG***GATTTCC***T (Kpn I) -3’
Site-directed mutagenesis	SUZ12	L: SUZ12p1-MT	-91~ -84	F: ***ACTGTAC***CGCGAATTCAGTTAA
		L: SUZ12p2-MT	-1395 ~ -1388	F: AGGCTTCA***GTACGGC***TGAGAA
		L: SUZ12p3-MT	-91~ -84 and -1395 ~ -1388	F: ***ACTGTAC***CGCGAATTCAGTTAA and AGGCTTCA***GTACGGC***TGAGAA
	CCDC43	L: CCDC43p1-MT	-737~ -714	F: TTGTTTGTTTTGAGAC***GTAGTCT***
		L: CCDC43p2-MT	-1389 ~ -1369	F: GAAGCCTTCCTG***GGTCTTC***T
		L: CCDC43p3-MT	-737~ -714and -1389 ~ -1369	F: TTGTTTGTTTTGAGAC***GTAGTCT*** and GAAGCCTTCCTG***GGTCTTC***T
Chip	SUZ12	Chip 1	L: -179~ -155	CTAAGGATCTAGACTCGCTAAACC
			R: 92 ~ 77	GATTCCCCCGTCAGTCAC
		Chip 2	L:-1477 ~ -1457	GTGCCCCAAGAGCTTAACAG
			R:- 1296~ -1272	CAGGCACTGCTAACACTACTATGA
	CCDC43	Chip 1	L: -757~ -737	CCTGGCTTTTGTTGTTGTTG
			R: -564 ~ -544	GGAGAACCCCCGTCTCTACT
		Chip 2	L:-1404 ~ -1384	CATCATCTCCACTGGGAAGC
			R:- 1228~ -1204	TTTTTAATCCATTAGGAACAGACA

*Bold and *italic*: HMGA1 binding sites; underline: mutated *nucleotide* residues.

Supplementary Table 2. Correlation between HMGA1 protein expression and the clinicopathological parameters of gastric carcinoma.

Features	Total number (n=51)	HMGA1 expression		*P*
		Low	High	
Age (years)				
<60	30	10(33.3%)	20(66.7%)	0.249
>60	21	11(52.4%)	10(47.6%)	
Gender				
Male	34	15(44.1%)	19(55.9%)	0.764
Female	17	6(35.3%)	11(64.7%)	
Differentiation				
Well	11	8(72.7%)	3(27.3%)	0.028
Moderate	16	7(43.8%)	9(56.2%)	
Poor	24	6(25.0%)	18(75.0%)	
Lymph node metastasis				
Yes	36	10(27.8%)	26(72.2%)	0.004
No	15	11(73.3%)	4(26.7%)	
Tumor size(cm^3)				
<5	27	16(59.3%)	11(40.7%)	0.009
≥ 5	24	5(20.8%)	19(79.2%)	
AJCC stage				
T1,T2	11	8(72.7.6%)	3(27.3%)	0.035
T3,T4	40	13(32.5%)	27(67.5%)	
AJCC TNM stage				
I`II	16	12(75.0%)	4(25.0%)	0.002
III`IV	35	9(25.7%)	26(74.3%)	
